# Supplementary material for: Feeding behaviour in a ‘basal’ tortoise provides insights on the transitional feeding mode at the dawn of modern land turtle evolution
Source: PeerJ. 2015 Aug 11;3:e1172. doi: 10.7717/peerj.1172 (PMC4558077; doi:10.7717/peerj.1172)
Supplement: Appendix S1 [file peerj-03-1172-s001.doc]

**APPENDIX**

List of die abbreviations in the text in alphabetic order:

C – Carapax rostral tip;

cm – Centimetre;

dors. – Dorsal;

FC – Fast close;

FU – Food uptake;

g – Gram;

GCM – Generalised Cyclic Model;

HP – Head protraction;

HPR – Head protraction;

HR – Head retraction;

Hy – Position of the basis of ceratbranchial I;

HyDD – Hyoid dorsal displacement;

HyRV – Hyoid retraction velocity;

HyVD – Hyoid ventral displacement;

LJ – Lower jaw tip;

MG – Maximum gape;

mm – Millimetre;

P – Point on the supraoccipital caudal end;

PG – Peak gape;

pro. – Protraction;

retr. – Retraction;

s – Second;

SO – Slow open phase lacking discrete SOI and SOII;

SO I – Slow Open I;

SO II – Slow Open II;

T – Transport;

TCD – Total cycle duration;

THC – Total hyoid cycle;

TP – Tongue plateau;

TPG – Time to Peak Gape;

TPR – Tongue protraction;

TR – Tongue retraction;

TT – Tip of the tongue;

Tv – Tympanim venral most point;

UJ – Upper jaw tip;

vent. – Ventral;
